# Knowledge base toward understanding actionable alterations and realizing precision oncology

**DOI:** 10.1007/s10147-018-1378-0

**Published:** 2018-12-12

**Authors:** Shiho Takeuchi, Shujiro Okuda

**Affiliations:** 1 Division of Cancer Genome Informatics, Niigata University Graduate School of Medical and Dental Sciences, 1-757 Asahimachi-dori, Chuo-ku, Niigata, 951-8510 Japan; 2 Division of Bioinformatics, Niigata University Graduate School of Medical and Dental Sciences, 1-757 Asahimachi-dori, Chuo-ku, Niigata, 951-8510 Japan

**Keywords:** Knowledge base, Next-generation sequencing, Precision oncology

## Abstract

In Japan, the National Cancer Center and university hospitals have initiated next-generation sequencing-based in vitro diagnostic testing for cancer patients as a method of clinical sequencing. Based on the molecular alterations detected, physicians can provide approved targeted therapy and access to investigational drugs for cancer patients. However, interpretation of the clinical significance of genomic alterations remains the most severe bottleneck of precision medicine in cancer. Although many research institutes in the United States are developing knowledge bases for interpretation of the tumor alterations and clinical decisions, these knowledge bases are unsuited as sources of reference in Japan due to differences in the information on approved drugs and implementation of clinical trials. In this review, we introduce knowledge bases for clinical decision-making based on genomic events in cancer, and discuss the resources of additional information necessary for implementing precision medicine in Japan.

## Introduction

Cancer is a disease caused by genetic mutation, with common mutations that occur across cancer types yet vary among individual patients. Traditional cancer treatment involves the selection of therapy that is specific for each type of cancer. With the advent of precision medicine, a more effective treatment strategy is implemented according to the genetic profile of each patient’s cancer.

For example, the EGFR gene mutation is found in approximately half of Japanese lung adenocarcinomas [1, 2] and is an important marker for treatment selection. When the EGFR mutation is found in tumor cells of a lung cancer, gefitinib [4, 5], erlotinib [6], and afatinib [7–10], drugs that target this mutation, are used as successful treatments. However, when the EGFR mutation is T790M, the tumor shows resistance to the above drugs [11–15], and osimertinib, which has a different mechanism of action, is a better treatment option [16]. In addition, when gene amplification of another tyrosine kinase such as MET is observed, resistance to EGFR inhibitors is predicted by bypass signaling [17, 18]. In another example, colorectal cancer patients with constitutively active mutations in the KRAS gene are unlikely to benefit from the EGFR inhibitors cetuximab and panitumumab [19–21].

Thus, the most rational therapeutic agent can be selected by considering the type of genetic mutation, the change in functionality due to the mutation, and the signal transduction pathways involved.

Therefore, it is necessary to detect mutations for multiple genes simultaneously, and then to curate clinically meaningful mutations that can be targeted for treatment, among many mutations. In this review, we discuss the knowledge base for efficiently collecting reference information necessary for the curation process that is essential in the practice of precision oncology.

## Comprehensive cancer panel sequencing tests

Gene panel testing is a technique that simultaneously analyzes multiple genes using next-generation sequencing (NGS). The gene panel testing methods currently used for cancer diagnosis can be roughly grouped into two types: companion diagnostics (CDx), and other in vitro diagnostics (IVD). The former is used to assess the treatment outcome of administering a specific therapeutic agent that had been authorized for a cancer with a specific genetic profile, and thus to decide whether to administer that drug as a treatment for that specific cancer. Companion diagnostics use predictive biomarker assays that guide the use of targeted cancer drugs, and are often developed in parallel to the drug [22]. On the other hand, gene profiling obtained with the latter provides information on the diagnosis or prognosis, and assists in the determination of the potential therapeutic strategy including the molecular-targeted drug. Such treatment regimens include approved drug therapy for specific tumor types, off-label treatment for unapproved tumor types, and clinical trials with investigational drugs.

Table 1 shows genetic panel tests currently being conducted in Japan (including tests for research purposes). In April 2018, the Oncomine™ Dx Target Test CDx system was approved as the first gene panel testing in Japan. The Oncomine™ Dx Target Test approved in the United States analyzes 46 target genes, and has been approved as a CDx to determine whether to administer the Food and Drug Administration (FDA)-approved drugs targeting three genes (*EGFR, BRAF*, and *ROS1*) among 23 genes related to non-small-cell lung cancer (NSCLC) [23]. In Japan, the same system was approved as a CDx to determine treatment only for the BRAF (V600E) mutation (http://www.info.pmda.go.jp/ygo/pack/840863/23000BZX00089000_A_01_01/).

**Table 1:** Comprehensive cancer panel sequencing tests for implementation in Japan

Comprehensive cancer panel sequencing tests	Target			Approval
	DNA	RNA	Fusion	
Oncomine™ Dx Target Test CDx system	1 (46)^a^	(46)^a^		Authorized by PMDA as a CDx for BRAF V600E ^b^
NCC OncoPanel	114		13	The Japanese Advanced Medical Care B program
Todai OncoPanel	465	467	17	The Japanese Advanced Medical Care B program
Oncomine™ Target Test	46	46		The Japanese Advanced Medical Care B program
OncoPrime	223			Research
MSK-IMPACT	468		18	Research
PleSSision	160			Research
Guardant360	73			Research
CANCERPLEX	435			research
ACTOnco+	440			Research
FoundationOne CDx™	324			Research (authorized by FDA as a CDx^c^)
Oncomine™ Dx Target Test	23 (46)^a^	23 (46)^a^		Research (authorized by FDA as a CDx^d^)
MSK-IMPACT	468		18	Research (authorized by FDA as an IVD^e^)

^a^Oncomine Dx target test and Oncomine Dx target test CDx system potentially detect alterations in 46 genes

^b^ http://www.info.pmda.go.jp/ygo/pack/840863/23000BZX00089000_A_01_01/

^c^ https://www.accessdata.fda.gov/cdrh_docs/pdf17/P170019A.pdf

^d^ https://www.accessdata.fda.gov/cdrh_docs/pdf16/P160045A.pdf

^e^ https://www.accessdata.fda.gov/cdrh_docs/pdf17/DEN170058.pdf

Meanwhile, the “NCC OncoPanel” provided by the National Cancer Center, the “Todai OncoPanel” provided by the University of Tokyo, and the “Oncomine Target Test” provided by Osaka University are being implemented as IVD options used for cancer genome medical treatment as of November 2018. The clinical usefulness of the above implementations is now being verified through the Japanese Advanced Medical Care B program of the Ministry of Health, Labour and Welfare (MHLW), with the aim of obtaining approval by the Pharmaceuticals and Medical Devices Agency (PMDA), and being implemented under insurance reimbursement of MHLW by 2019.

## Scheme for cancer genome medicine

**Fig. 1:**
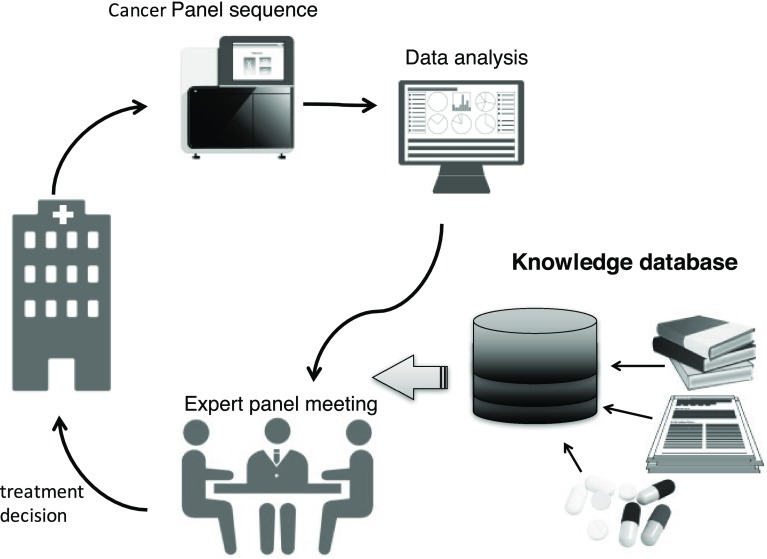
Scheme for cancer genome panel test. Genomic DNA is extracted from tumor tissue or biopsies, and sequenced. Variants are called annotated and prioritized for potential functional or clinical relevance before being reported to a tumor board, where an interdisciplinary team “expert panel” decides the treatment options. The knowledge base contains useful information on evidence in literature, clinical research, therapeutic drugs, etc.

Unlike CDx, determining the outcome in IVD tests is complicated. Figure 1 shows the analysis scheme of cancer genome medical treatment in Japan. Sequence information obtained by genetic panel testing is converted into mutation information after bioinformatics analysis. Subsequently, a curation process is performed, which specifies clinically useful mutations such as pathogenic mutations leading to treatment, followed by the preparation of a report. Finally, an expert panel consisting of experts from several fields decides the treatment policy taking into consideration existing adaptable medicines and participable clinical trials. Depending on the implementing facility, members of the expert panel, who are required to have extensive knowledge and highly advanced skills, may be responsible for the curation step. Furthermore, the reference information and the curation methods used are different in each facility, so the final interpretation may be different depending on the implementation facility. Therefore, for the purpose of equalizing the quality and standardizing the process of curation as much as possible, it would be necessary to collect information mechanically.

## Knowledge base for curation and treatment selection

Table 2 lists the knowledge bases for mechanically collecting necessary information for the curation process. There are different types of knowledge bases. One is a database that aggregates meaningful mutations among genetic mutations obtained from NGS data. The other is a knowledge base that automatically integrates information on treatments and clinical trials, and performs evidence-level classification based upon this information. COSMIC [24], which accumulates data specifically on somatic mutations of human cancer, and ClinVar [25], which is specialized for pathological mutations, are databases of the former type. In the ClinVar database, the meaning of mutation may be inconsistent because it is defined by the registrant. In addition, ClinGen is a database that performs a curation process with uniform standards, and redefines mutations [26–29].

**Table 2:** Knowledge base for curation and treatment decision

Databases	Primary institute	Variant	Treatment	Clinical trials	URL
COSMIC	Wellcome Trust Sanger Institute	Somatic			http://cancer.sanger.ac.uk/cosmic
ClinVar	National Center for Biotechnology Information (NCBI)	All variants			http://www.ncbi.nlm.nih.gov/clinvar/
ClinGen Knowledge Base	ClinGen	All variants			https://www.clinicalgenome.org/resources-tools/
Cancer Genome Interpreter (CGI)	Institute for Research in Biomedicine, Barcelona, Spain	Somatic	Yes		https://www.cancergenomeinterpreter.org/home
Clinical Interpretation of Variants in Cancer (CIViC)	Washington University School of Medicine (WashU)	All variants	Yes		http://www.civicdb.org
JAX Clinical Knowledgebase (JAX-CKB)	The Jackson Laboratory	Somatic	Yes	Yes	https://ckb.jax.org/
OncoKB	Memorial Sloan Kettering Cancer Center	Somatic	Yes		http://oncokb.org/#/
Precision Medicine Knowledgebase (PMKB)	Weill Cornell Medical College	Somatic			https://pmkb.weill.cornell.edu/
Cancer Driver Log (CanDL)	Ohio State University (OSU)/James Cancer Hospital	Somatic	Yes		https://candl.osu.edu/
My Cancer Genome	Vanderbilt University	Somatic	Yes	Yes	https://www.mycancergenome.org/
DoCM	Washington University	All variants			http://docm.info
Personalized Cancer Therapy Database	MD Anderson Cancer Center	Somatic	Yes	Yes	https://pct.mdanderson.org

Integrated knowledge bases include OncoKB [30] developed for MSK-IMPACT panel analysis, JAX Clinical Knowledgebase (JAX-CKB) [31] provided by The Jackson Laboratory, Precision Medicine Knowledgebase (PMKB) [32], Cancer Driver Log (CanDL) [33], My Cancer Genome [34], Personalized Cancer Therapy Database [35], Cancer Genome Interpreter (CGI) [36], DoCM [37], CIVic [38–40], and others.

OncoKB is a curated database for MSK-IMPACT panel analysis. It has annotated 4232 alterations in 554 genes, 38 tumor types, and 76 drugs. OncoKB contains information on alteration types, and provides information on the actionability and therapeutic implications, classified into the following six levels according to available evidence: Level 1, FDA approved; Level 2, Standard care; Level 3, Clinical evidence; Level 4, Biological evidence; Level R1, Standard care resistance; and Level R2, Clinical evidence of resistance. OncoKB provides multiple-access methods, including an application programming interface (API), data file download, and web browser-based data access, while information on clinical trials is not included.

The JAX-CKB database contains gene and variant descriptions, drug indication status, clinical trials by indication, treatment approaches, efficacy evidence supporting response to treatment approaches by indication, and resistance evidence supporting resistance to treatments by indication. Since October 2018, the Jackson Laboratory has introduced a new tiered structure in which users may choose between three levels of access: CKB Core (TM) provides free access; CKB Boost (TM), the newest option, provides only web-access to 1000 + genes; and CKB Flex (TM) provides scalable and flexible content integration into bioinformatic workflows for these 1000 + genes.

The Public Access version of JAX-CKB contains only 82 commonly known driver genes. Users can search JAX-CKB for genes, genetic mutants, drugs, drug classes, indications, and clinical trials. The web-based version of JAX-CKB is designed to query the knowledge base for specific data attributes.

PMKB provides information about clinical cancer variants and interpretations in a structured way, as well as allowing users to submit and edit existing entries. The database contains 457 variant descriptions with 281 clinical-grade interpretations. Importantly, all interpretations are either written or approved by certified molecular pathologists.

CanDL is an expert-curated database for actionable driver mutations, which has recorded 373 variants in 60 genes. These mutations are categorized into four groups depending on the evidence level and published literature as follows: mutations corresponding to FDA approved or The National Comprehensive Cancer Network recommended therapy; mutations with treatment based on evidence from clinical trials, case reports, or exceptional responders; mutations that can predict response or resistance based on evidence in pre-clinical data (in vitro or in vivo); and putative oncogenic driver mutations. The CanDL database can be searched by genetic unit or amino acid substitution, and allows users to download all or selected genes as a comma-separated value file. However, the latest data update is July 2015 according to the official website.

My Cancer Genome contains an overview of mutations, actionability, therapeutic implications, and available clinical trials. In addition to a web interface, My Cancer Genome provides access via API and a Mobile App to facilitate dissemination of information and enhance accessibility. The information in My Cancer Genome is hierarchically organized by cancer types, cancer-related genes, and specific cancer-related genetic mutations. However, alteration-specific information is organized by tumor type rather than by alteration, which may cause difficulties when extrapolating information for adaptation to other tumor types.

Personalized Cancer Therapy Database is a semi-public knowledge base provided by MD Anderson Cancer Center. This knowledge base provides information on the function of common genomic alterations and their therapeutic implications for about 32 genes important in cancer. The contents of this database include overviews on genes and their function, genetic alterations, frequencies and outcomes, therapeutic implications, FDA-approved drugs or investigational therapeutics in clinical trials targeting a pathway, and genotype-selected clinical trials and genotype-relevant clinical trials. In addition to mechanically obtained information, stored information is reviewed manually by experts from multiple disciplines, and especially, information from clinical trials is updated weekly. Information is organized for each gene regardless of the tumor type, while it is also possible to search for each mutation. However, the API for this database is not disclosed at this time.

CGI is the knowledge base with a reference database and curation system. CGI collects known oncogenic alterations in a tumor, and predicts the effect of the remaining alterations of uncertain significance. It also reports the known influence of these variants on drug response according to the level of supporting clinical evidence, and lists the interactions of existing chemical compounds with genes bearing driver alterations. While freely available through an API or a web interface, information resources in CGI include the Catalog of Cancer Genes, Catalog of Validated Oncogenic Mutations, Cancer Biomarkers database, and Cancer Bioactivities Database.

DoCM assembles known pathogenic variants validated in cancer. DoCM contains information on 122 cancer sub-types, 132 genes, 1364 variants, variant types, variant effects, and evidence from the literature. DoCM is provided as an open source and open license database, and the API system for data download has been released.

CIViC summarizes and aggregates evidence of clinically actionable variants into clinical interpretations, and is based on crowdsourcing. As a result, CIViC is a highly transparent knowledge base with all information from the source of evidence to the source code open and accessible. Furthermore, DoCM and CIVic share complementary information.

## Approved drug database

Although many useful knowledge bases have been developed in Europe and the United States, information on therapeutic drugs and related evidence is based on FDA- and European Medicines Agency (EMA)-approved drugs, and trial information in Europe and the United States. For the implementation of precision medical treatment in Japan, verification with approved drugs in Japan and clinical trial information corresponding to the detected genetic variation are essential. Therefore, it is necessary to collect and refer to drug information with PMDA approval as shown in Table 3.

**Table 3:** Approved drug database

Region/country/agency	Database	Operating organization	URL
United States	Drugs@FDA	FDA/CDER	https://www.accessdata.fda.gov/scripts/cder/daf/
United States	A to Z List of Cancer Drugs	NCI	https://www.cancer.gov/about-cancer/treatment/drugs?redirect=true
European Union	EPAR (European Public Assessment Reports)	EMA	https://www.ema.europa.eu/en/medicines/field_ema_web_categories%253Aname_field/
Japan	Search system for Prescription Drugs (in Japanese)	PMDA	http://www.pmda.go.jp/PmdaSearch/iyakuSearch/

*FDA* Food and Drug Administration, *CDER* Center for Drug Evaluation and Research, *NCI* National Cancer Institute, *EMA* European Medicines Agency, *PMDA* Pharmaceuticals and Medical Devices Agency

## Clinical trial information

In order to determine whether clinical studies are available for patients without indication for standard therapy, it is necessary to collect information on clinical research. A database that collects information on clinical research in each country is shown in Table 4. There is a centralized database on clinical trials in the United States, because there is an obligation to register clinical trial implementation with the FDA. In Europe, apart from each national trial, there is a central application in the EU, which is managed centrally by the EMA. In Japan, the Japan Primary Registries Network (JPRN), which integrates information from three organizations—the University hospital Medical Information Network (UMIN) clinical trial registration system (UMIN-CTR), Japan Medical Information Center (JAPIC), and Japan Medical Association Center for Clinical Trials (JMACCT)—was established and internationally recognized as a primary registry in 2008. JPRN provides clinical test information to the International Clinical Trial Registry Platform (ICTRP) managed by the World Health Organization (WHO). International Committee of Medical Journal Editors (ICMJE) requires that information be registered in WHO’s ICTRP primary registry as a condition for publication. However, in Japan, registration with the three organizations has been optional thus far, and all associated information was not always disclosed. In April 2018, the Clinical Trials Act was enacted, which mandated the registry of clinical trial implementation plans via the clinical research implementation plan/research outline release system, Japan Registry of Clinical Trials (jRCT). It is expected that information on the clinical trials conducted in Japan will be more efficiently and comprehensively collected when this system is in operation.

**Table 4:** Clinical trial databases (administrative organ)

Region/country/agency	Database	Operating organization	URL
United States	ClinicalTrials.gov^a^	FDA	https://clinicaltrials.gov
European Union	EudraCT (The European Clinical Trials Database)	EMA	https://eudract.ema.europa.eu/index.html
European Union	The EU Clinical Trials Register^a^	EMA	https://www.clinicaltrialsregister.eu/ctr-search/search
United Kingdom	ISRCTN registry^a^	HRA	http://www.isrctn.com
Japan (current system)	JPRN (Japan Primary Registries Network)^b^ Network members		
	UMIN-CTR (UMIN Clinical Trials Registry)	UMIN	http://www.umin.ac.jp/ctr/index.htm
	JAPIC-CTI (JAPIC Clinical Trials Information)	JAPIC	http://www.clinicaltrials.jp/user/cteSearch_e.jsp
	JMACCT-CTR (JMACCT Clinical Trial Registry)	JMACCT	http://www.jmacct.med.or.jp/en/what-we-do/registry.html
	NIPH Clinical Trials Search^a^	NIPH	https://rctportal.niph.go.jp
Japan (latest system)	j RCT (Japan Registry of Clinical Trials)	NIPH	https://jrct.niph.go.jp
United Nations	ICTRP (International Clinical Trials Registry Platform)	WHO	http://apps.who.int/trialsearch/

*EMA* European Medicines Agency, *EORTC* European Organisation for Research and Treatment of Cancer, *FDA* Food and Drug Administration, *HRA* Health Research Authority, *ICMJE* International Committee of Medical Journal Editors, *JAPIC* Japan Pharmaceutical Information Center, *JMACCT* Center for Clinical Trials, Japan Medical Association, *NIPH* National Institute of Public Health, *UMIN* University hospital Medical Information Network, *WHO* World Health Organization

^a^Registries that are recognized by the International Committee of Medical Journal Editors

^b^Cross-search of the content of three national clinical research information registries: UMIN-CTR, JAPIC-CTI, and JMACCT-CTR

## Collateral of curation quality, and standardization and sharing of methods

In April 2018, the US FDA issued two guidance documents on NGS-based tests [41, 42]. The knowledge base that collects evidence supporting the association between a genetic variant and disease was institutionalized to allow it to be open access with the FDA approval system. To obtain certification, it is necessary to perform rigorous examination of application accuracy, standard operation procedures (SOPs), security, information management, etc. These public databases are expected to contain resources similar to those of ClinGen [28]. This system provides the developers of NGS-based genetic examinations the advantage of reduced burden from additional clinical data by supporting the use of the FDA-approved database. While the draft guideline published in 2016 pertained to germline mutations, the final version also included somatic mutations.

Thus, the government of the United States is working to ensure that quality beyond a certain level is guaranteed. Furthermore, dissemination is promoted by ensuring that the databases are open source. Developers of the knowledge bases also organized the Variant Interpretation for Cancer Consortium (VICC) as the driver project of the Global Alliance for Genomics Health (GA4GH), and developed meta-knowledgebase (https://search.cancervariants.org/) that can perform curation across databases. The beta version of this project has been released [43].In Japan, no consensus method has been obtained for curation thus far. In each facility, both the reference database used for curation and the curation method are different, and it is often not publicly available. “Clinical practice guidance for next-generation sequencing in cancer diagnosis and treatment (Edition 1.0)” [44] was published by Japanese Society of Medical Oncology, Japan Society of Clinical Oncology, and Japan Cancer Association. The accompanying “Evidence Levels of Gene Panel Testing Results (ver. 1.0) as of Aug 21, 2017” is a document that is helpful for selecting treatment methods that can be implemented in Japan. However, since the schedule for information update is undecided, other sources of information will be also needed in the near future to obtain approval information on drugs, which is changing on a daily basis, as well as the latest clinical trial information.

## Conclusion

Despite many challenges as discussed above, cancer genome therapy is utilized in Japan. To select an effective treatment method from the gene profile of cancer cells of a patient, curation work is indispensable in identifying the clinical significance of the mutation information obtained, and whether the applicable therapy exists or not. To conduct high-quality curation, it is necessary to comprehensively collect a wide variety of abundant information, continue updating information on drugs and clinical trials that are constantly updated on a daily basis, and link the latest information correctly. Therefore, the development of an integrated knowledge base system, which takes advantage of bioinformatics and automatic curation, while integrating the existing knowledge base with information on proprietary approved drugs and clinical trials, is urgently needed, and expected to be implemented in the near future, especially in Japan.
